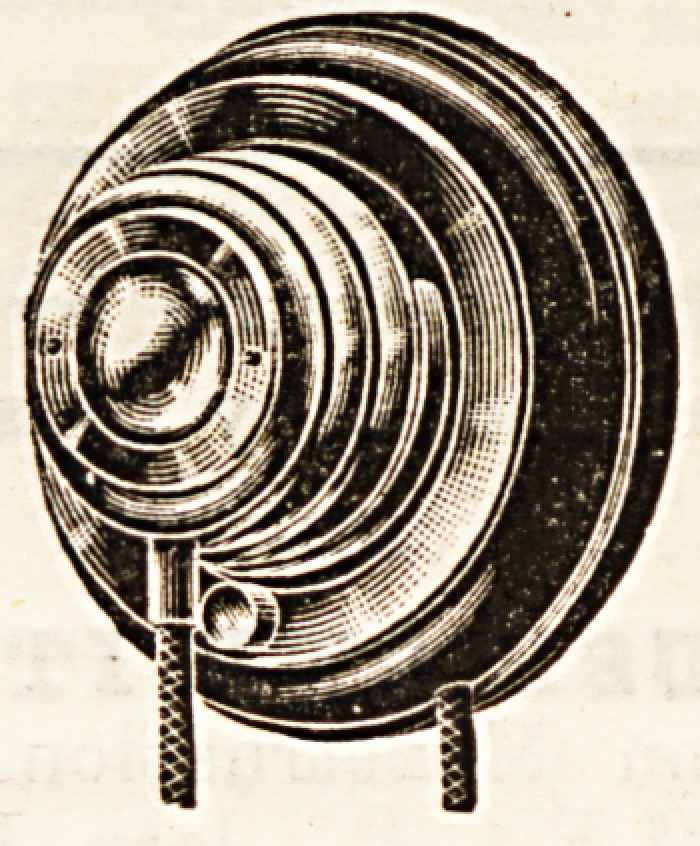# Practical Departments

**Published:** 1897-11-27

**Authors:** 


### PRACTICAL DEPARTMENTS.

### THE "DIAMOND JUBILEE" PORTABLE HOSPITAL TELEPHONE.

(London and Provincial Telephone Company, 11, Queen Victoria Street, London, E.C.)

This is introduced as a ready and convenient means of establishing communication between rooms in which infectious diseases are being isolated and the outside world. It certainly is a very efficient apparatus for the purpose, and is thoroughly portable. The advantage of having telephonic communication with a sick-room is so obvious that little need be said about it. The most striking benefit of such an arrangement is seen in connection with infectious disease. In such cases the isolation can be made much more perfect by the nurse using the telephone instead of explaining her wants by word of mouth; while, when the convalescent stage is reached, it must often be a most pleasant and interesting thing for the relatives now and again to hold converse with the isolated invalid. We do not think, however, that the advantage of these portable telephones is at all confined to cases of infectious disease. Wherever active nursing is required it becomes necessary to maintain the rule that the nurse shall not leave the sick-room to fetch things, and there is no doubt that one of the great causes of the unpopularity of trained nurses among the servants arises from their constantly ringing the bell in obedience to this rule. A telephone, then, will tend greatly to oil the wheels of the domestic machine by saving the poor housemaid many a useless climb into the upper storeys. The apparatus consists of a transmitter and receiver at each end, with twelve yards of connecting wire cord. The battery is enclosed in the case, so that there are no loose parts, and no fixing is required. One box is placed on a table or a chest of drawers in the sick-room, and the other in any convenient support in the room to be communicated with, the wire cords are laid on or under the carpet, passing easily under the doors, and the arrangement is complete. Instead of ringing a bell, pressing a button sets up a gentle rattle, which is quite enough to call attention. The price of the two instruments complete, with twelve yards of wire cord and connecting pins, is £4 10s., and an extra twelve yards of wire cord with connecting pins to extend the distance can be had for another 6s. We have tested the apparatus and have proved it to be perfectly efficient.

We would also draw special attention to another form of the apparatus which has been made for use in houses which are fitted with electric bells. This form is very cheap, only costing 36s. 6d. for the two instruments, and is arranged to fix on to the electric bell wire, one end speaking in the bedroom and the other in the kitchen or wherever the bell is made to ring. The existing battery and bell wires are left intact, and all that is required is to substitute one of the telephones for the bell push and fix the other to the indicator, which can easily be done by any local electric bell fitter. As a means of communicating between the nurse and the servants this will be found a most useful arrangement, but its utility may clearly be extended to many other circumstances besides sickness.

### INVALID FURNITURE.

Our attention has been drawn to the new department for invalid furniture opened recently at 7, Parkside, Knightsbridge, by Messrs. Leveson, of Oxford Street. For some time this firm has had a show-room for baby carriages, and it has been felt that larger and more convenient premises would be an advantage both to the firm and its customers. The repairing workshops on the new premises, are a convenience that the residents in the neighbourhood will find useful, enabling customers to have repairs done in an hour or so, which would take as many days if sent to head-quarters. There are many contrivances for the comfort of invalids in the shape of couches, chairs, tables, &c. The combined Merlin and carrying chair is very ingenious, and patients who have to be carried from one room to another up and down stairs will find it most useful. It would be a great convenience also at railway stations; the chair could be lifted into the carriage, the patient placed in it, and both lifted out together, put upon the "Merlin" frame, and wheeled away. The bedside chair is a novelty also; one arm turns right back on hinges, and enables the patient to be moved from the bed to the chair comfortably, and then the arm is replaced. The back, seat, and foot rest is adjustable, and the position of the patient altered at will by means of cranks. A patient lifter for the use of hospitals is worthy of note. The canvas sheet is inserted under the patient with the draw sheet, the elevator frame is then placed in position, and the sheet attached by means of cranks, the patient is then lifted some feet above the bed, enabling the mattress to be removed or turned and the bed made before he is lowered into it, the detachment is then made; the canvas sheet removed, and the frame wheeled off for further service. There are a large number of "Merlin" (*i.e.*, wheel chairs which patients can themselves propel) chairs of varying prices, and which have found much favour amongst nurses. There are also many kinds of bath chair, from the elaborate adjustable spinal carriage to the simple basket frame on ordinary wheels, as well as the usual complement of ordinary nursing necessities.

## Figures and Tables

**Figure f1:**
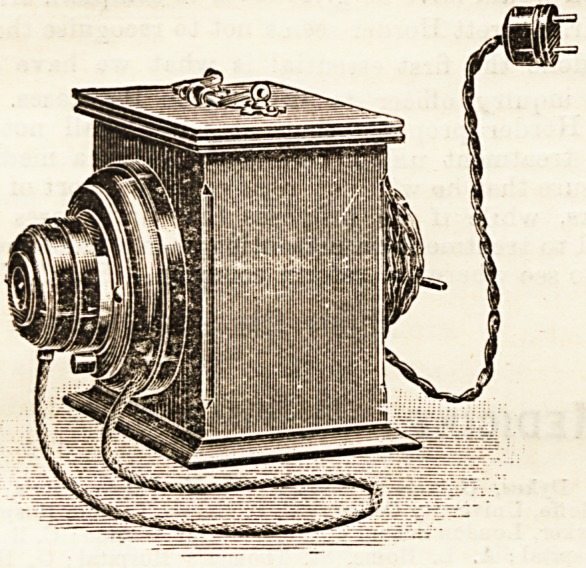


**Figure f2:**